# Supplementary material for: Prognostic Value of the Time-to-Positivity in Blood Cultures from Septic Shock Patients with Bacteremia Receiving Protocol-Driven Resuscitation Bundle Therapy: A Retrospective Cohort Study
Source: Antibiotics (Basel). 2021 Jun 8;10(6):683. doi: 10.3390/antibiotics10060683 (PMC8228862; doi:10.3390/antibiotics10060683)
Supplement: Supplementary file 1 [file antibiotics-10-00683-s001.zip › antibiotics-1215632-supplementary.pdf]

Table S1. Baseline characteristics of the septic shock patients with *Escherichia coli* bacteremia

Characteristics	Total (N = 333)	Survivor (N = 296)	Non-survivor (N = 37)	P-value
Age	67.0 (59.0 – 75.0)	68.0 (59.0 – 76.0)	66.5 (56.8 – 80.0)	0.50
Male	169 (50.8)	147 (49.7)	22 (59.5)	0.26
Past illness				
HTN	110 (33.0)	97 (32.8)	13 (35.1)	0.77
DM	90 (27.0)	78 (26.4)	12 (32.4)	0.43
CAD	31 (9.3)	26 (8.8)	5 (13.5)	0.35
Pulmonary disease	16 (4.8)	14 (4.7)	2 (5.4)	0.85
Malignancy	164 (49.2)	142 (48.0)	22 (59.5)	0.19
Hematologic disorder	18 (5.4)	16 (5.4)	2 (5.4)	1.00
CKD	25 (7.5)	20 (6.8)	5 (13.5)	0.14
Site of infection				
Unknown	14 (4.2)	12 (4.1)	2 (5.4)	0.69
Lung	22 (6.6)	12 (4.1)	10 (27.0)	< 0.01
Urinary tract	95 (28.5)	91 (30.7)	4 (10.8)	0.01
Intra-abdomen	27 (8.1)	22 (7.4)	5 (13.5)	0.20
Hepato-biliary-pancreas	157 (47.1)	144 (48.6)	13 (35.1)	0.12
Others	9 (2.7)	8 (2.7)	1 (2.7)	0.66
Initial vital signs				
SBP (mmHg)	94.0 (78.0 – 111.0)	90.0 (80.0 – 107.5)	93.5 (71.3 – 107.5)	0.54
DBP (mmHg)	58.0 (49.5 – 70.0)	58.0 (50.0 – 67.0)	56.0 (39.8 – 71.0)	0.74

PR (/min)	110.0 (95.0 – 125.5)	109.0 (94.5 – 126.5)	117.0 (104.3 – 125.0)	0.47
RR (/min)	20.0 (20.0 – 22.0)	20.0 (18.0 – 20.0)	22.0 (20.0 – 24.0)	< 0.01
BT (℃)	38.2 (37.2 – 39.2)	38.2 (37.5 – 39.2)	37.9 (37.1 – 39.1)	0.39
Laboratory				
WBC (×103/μL)	8.4 (4.2 – 14.6)	8.1 (4.2 – 14.0)	5.9 (1.2 – 19.1)	0.08
Hemoglobin (g/dL)	11.0 (9.0 – 12.5)	11.0 (9.3 – 12.5)	8.9 (6.8 – 12.1)	< 0.01
PT (INR)	1.3 (1.1 – 1.4)	1.2 (1.1 – 1.4)	1.4 (1.2 – 1.9)	0.01
Lactate (mmol/L)	3.4 (2.0 – 5.9)	3.2 (1.8 – 5.9)	5.1 (3.3 – 11.2)	< 0.01
BUN (mg/dL)	25.0 (16.5 – 35.0)	25.0 (17.5 – 35.0)	29.5 (22.0 – 39.3)	0.02
Creatinine (mg/dL)	1.3 (1.0 – 2.0)	1.3 (0.9 – 1.9)	1.3 (1.1 – 2.7)	0.05
Total bilirubin (mg/dL)	1.5 (0.8 – 3.1)	1.6 (0.9 – 3.1)	1.4 (0.6 – 1.9)	0.35
CRP (mg/dL)	11.0 (4.3 – 19.3)	10.6 (3.8 – 20.7)	12.6 (6.6 – 28.3)	0.22
SOFA score	7.0 (5.5 – 10.0)	7.0 (5.0 – 10.0)	9.5 (7.0 – 13.3)	< 0.01
TTP (hours)	9.5 (7.8 – 11.4)	9.8 (8.2 – 11.9)	8.6 (7.2 – 9.3)	0.01
Interventions				
Source control	159 (47.7)	142 (48.0)	17 (45.9)	0.82
Antibiotics escalation	57 (17.1)	52 (17.6)	5 (13.5)	0.54
ICU admission	160 (48.0)	138 (46.6)	22 (59.5)	0.14
Mechanical ventilator	48 (14.4)	30 (10.1)	18 (48.6)	< 0.01

RRT	45 (13.5)	26 (8.8)	19 (51.4)	< 0.01

Data are presented as a number (%) or as a mean with standard deviation.

Abbreviations: HTN = hypertension; DM = diabetes mellitus; CAD = coronary artery disease; CKD = chronic kidney disease; LC = liver cirrhosis; SBP = systolic blood pressure; DBP = diastolic blood pressure; PR = pulse rate; RR = respiratory rate; BT = body temperature; GCS = Glasgow Coma Scale; WBC = white blood cells; PT = prothrombin time; INR = international normalized ratio; BUN = blood urea nitrogen; CRP = C-reactive protein; SOFA = sequential organ failure assessment; TTP = time-to-positivity; ICU = intensive care unit; RRT = renal replacement therapy.

Table S2. Baseline characteristics of the septic shock patients with *Klebsiella* species bacteremia

Characteristics	Total (N = 191)	Survivor (N = 162)	Non-survivor (N = 29)	P-value
Age	65.0 (58.0 – 75.0)	68.0 (59.0 – 76.0)	66.5 (56.8 – 80.0)	0.51
Male	125 (65.4)	103 (63.6)	22 (75.9)	0.20
Past illness				
HTN	60 (31.4)	49 (30.2)	11 (37.9)	0.41
DM	56 (29.3)	45 (27.8)	11 (37.9)	0.27
CAD	13 (6.8)	8 (4.9)	5 (17.2)	0.02
Pulmonary disease	8 (4.2)	7 (4.3)	1 (3.4)	0.83
Malignancy	106 (55.5)	86 (53.1)	20 (69.0)	0.11
Hematologic disorder	9 (4.7)	8 (4.9)	1 (3.4)	0.73
CKD	20 (10.5)	16 (9.9)	4 (13.8)	0.53
LC	5 (2.6)	4 (2.5)	1 (3.4)	0.76
Site of infection				
Unknown	9 (4.7)	8 (4.9)	1 (3.4)	0.73
Lung	18 (9.4)	13 (8.0)	5 (17.2)	0.12
Urinary tract	15 (7.9)	12 (7.4)	3 (10.2)	0.59
Intra-abdomen	11 (5.8)	8 (4.9)	3 (10.3)	0.25
Hepato-biliary-pancreas	126 (66.0)	109 (67.3)	17 (58.6)	0.37
Others	5 (2.6)	5 (3.1)	0 (0.0)	0.46
Initial vital signs				
SBP (mmHg)	93.0 (80.0 – 112.0)	90.0 (80.0 – 107.5)	93.5 (71.3 – 107.5)	0.04

DBP (mmHg)	59.0 (49.0 – 71.0)	58.0 (50.0 – 67.0)	56.0 (39.8 – 71.0)	0.08
PR (/min)	115.0 (93.0 – 132.0)	109.0 (94.5 – 126.5)	117.0 (104.3 – 125.0)	0.61
RR (/min)	20.0 (20.0 – 22.0)	20.0 (18.0 – 20.0)	22.0 (20.0 – 24.0)	0.72
BT (℃)	38.2 (37.0 – 39.2)	38.2 (37.5 – 39.2)	37.9 (37.1 – 39.1)	0.02
Laboratory				
WBC (×103/μL)	8.3 (4.9 – 14.9)	8.1 (4.2 – 14.0)	5.9 (1.2 – 19.1)	0,46
Hemoglobin (g/dL)	10.8 (9.2 – 12.1)	11.0 (9.3 – 12.5)	8.9 (6.8 – 12.1)	0.14
PT (INR)	1.3 (1.2 – 1.5)	1.2 (1.1 – 1.4)	1.4 (1.1 – 1.9)	0.12
Lactate (mmol/L)	3.5 (2.2 – 5.6)	3.2 (1.8 – 5.9)	5.1 (3.3 – 11.2)	< 0.01
BUN (mg/dL)	22.0 (16.0 – 32.0)	25.0 (!7.5 – 35.0)	29.5 (22.0 – 39.3)	0.13
Creatinine (mg/dL)	1.2 (0.9 – 1.8)	1.3 (0.9 – 1.9)	1.3 (1.1 – 2.7)	0.04
Total bilirubin (mg/dL)	2.2 (1.0 – 3.9)	1.6 (0.9 – 3.1)	1.4 (0.5 – 1.9)	0.28
CRP (mg/dL)	13.8 (6.3 – 20.6)	10.6 (3.8 – 20.7)	12.6 (6.6 – 28.3)	0.94
SOFA score	8.0 (6.0 – 10.0)	7.0 (5.0 – 10.0)	9.5 (7.0 – 13.3)	0.01
TTP (hours)	9.4 (8.1 – 11.9)	9.8 (8.2 – 11.9)	8.6 (7.2 – 9.3)	0.03
Interventions				
Source control	99 (51.8)	88 (54.3)	11 (37.9)	0.10
Antibiotic escalation	27 (14.1)	23 (14.2)	4 (13.8)	0.95
ICU admission	94 (49.2)	79 (48.8)	15 (51.7)	0.77
Mechanical	37 (19.4)	25 (15.4)	12 (41.4)	< 0.01

ventilator				
RRT	25 (13.1)	16 (9.9)	9 (31.0)	< 0.01

Data are presented as a number (%) or as a mean with standard deviation.

Abbreviations: HTN = hypertension; DM = diabetes mellitus; CAD = coronary artery disease; CKD = chronic kidney disease; LC = liver cirrhosis; SBP = systolic blood pressure; DBP = diastolic blood pressure; PR = pulse rate; RR = respiratory rate; BT = body temperature; GCS = Glasgow Coma Scale; WBC = white blood cells; PT = prothrombin time; INR = international normalized ratio; BUN = blood urea nitrogen; CRP = C-reactive protein; SOFA = sequential organ failure assessment; TTP = time-to-positivity; ICU = intensive care unit; RRT = renal replacement therapy.

Table S3. Baseline characteristics of the septic shock patients according to the blood culture results.

Past illness	Total (N = 1718)	No bacteremia (N = 915)	Bacteremia (N = 803)	P-value
HTN	605 (35.2)	340 (37.2)	265 (33.0)	0.07
DM	425 (24.7)	223 (24.4)	202 (25.2)	0.71
CAD	182 (10.6)	103 (11.3)	79 (9.8)	0.34
Pulmonary disease	108 (6.3)	69 (7.5)	39 (4.9)	0.02
Malignancy	782 (45.5)	393 (43.0)	389 (48.4)	0.03
Hematologic disorder	86 (5.0)	56 (6.1)	30 (3.7)	0.02
CKD	118 (6.9)	68 (7.4)	50 (6.2)	0.32
LC	266 (15.5)	116 (12.7)	150 (18.7)	< 0.01

Data are presented as a number (%)

Abbreviations: HTN = hypertension; DM = diabetes mellitus; CAD = coronary artery disease; CKD = chronic kidney disease; LC = liver cirrhosis.

Table S4. Median TTP according to sites of infection.

Lung infection	Total (N = 109)	Survivor (N = 70)	Non-survivor (N = 39)	P-value
Median TTP (hr)	12.0 (9.2 – 18.8)	12.1 (9.3 – 20.4)	11.8 (9.0 – 15.9)	0.73

Urinary infection	Total (N = 143)	Survivor (N = 132)	Non-survivor (N = 11)	P-value
Median TTP (hr)	10.7 (8.6 – 15.1)	10.8 (8.8 – 15.0)	9.4 (7.4 – 21.1)	0.73

Hepato-biliary-pancreas infection	Total (N = 362)	Survivor (N = 322)	Non-survivor (N = 40)	P-value
Median TTP (hr)	9.8 (8.2 – 12.1)	9.9 (8.4 – 12.1)	9.0 (8.0 – 12.6)	0.35

Abbreviations: TTP = time-to-positivity.
